# *Schizosaccharomyces pombe* Can Reduce Acetic Acid Produced by *Baijiu* Spontaneous Fermentation Microbiota

**DOI:** 10.3390/microorganisms7120606

**Published:** 2019-11-22

**Authors:** Zhewei Song, Hai Du, Menghui Zhang, Yao Nie, Yan Xu

**Affiliations:** 1 State Key Laboratory of Food Science and Technology, Key Laboratory of Industrial Biotechnology of Ministry of Education, Synergetic Innovation Center of Food Safety and Nutrition, School of Biotechnology, Jiangnan University, Wuxi 214122, China; songzhewei@126.com (Z.S.); duhai88@126.com (H.D.); 2 State Key Laboratory of Microbial Metabolism, Joint International Research Laboratory of Metabolic & Developmental Sciences, Department of Microbiology, School of Life Sciences and Biotechnology, Shanghai Jiao Tong University, Shanghai 200240, China; mhzhang@sjtu.edu.cn

**Keywords:** non-*Saccharomyces* yeast, *Schizosaccharomyces pombe*, acetic acid reduction, mevalonate pathway, Chinese *Baijiu*, spontaneous fermentation, metatranscriptomic sequencing

## Abstract

The spontaneous fermentation of alcoholic beverage is a bioprocess donated by microbiota with complex stress environments. Among various microbes, non-*Saccharomyces* yeasts have high stress tolerance and significantly affect the taste and quality of products in process. Although many researchers have focused on the influence of acid stress, the mechanism of non-*Saccharomyces* yeasts to tolerant stress remains unclear in microbiota. To bridge the gap, we constructed in situ and in vitro studies to explore the reduction pathway of acetic acid in non-*Saccharomyces* yeasts. In this study, we found *Schizosaccharomyces pombe* has special capacities to resist 10 g/L acetic acid in laboratory cultures and decrease the average concentration of acetic acid from 9.62 to 6.55 g/kg fermented grains in Chinese Maotai-flavor liquor (*Baijiu*) production. Moreover, *Schi. pombe* promoted metabolic level of mevalonate pathway (high expressions of gene *ACCAT1*, *HMGCS1,* and *HMGCR1*) to degrade a high concentration of acetic acid. Meanwhile, *Schi. pombe* also improved the concentration of mevalonic acid that is the precursor of terpenes to enhance the taste and quality of *Baijiu*. Overall, the synchronicity of reduction and generation in *Schi. pombe* advances the current knowledge to guide more suitable strategies for mechanism studies of non-*Saccharomyces* yeasts in fermented industries of alcoholic beverages.

## 1. Introduction

The recent studies of modern biotechnologies have shown that non-*Saccharomyces* yeasts have significant effects which improve product quality in various alcoholic beverage industries [1,2,3]. With the developing of this field in the production, researchers have isolated and identified a serious of non-*Saccharomyces* yeasts including *Pichia kudriavzevii* [4], *Candida humilis* [5], *Saccharomycopsis fibuligera* [6], *Wickerhamomyces anomalus* [7], *Zygosaccharomyces bailii* [8], *Schizosaccharomyces pombe* [9], and *Torulaspora delbrueckii* [10] in microbiota. Moreover, the major functions of these non-*Saccharomyces* yeast have been discovered, such as acid regulation [2], higher alcohol reduction [11], and ester generation [12]. They can also decrease the concentrations of unsafe compounds that affect product quality, such as ethyl carbamate [13] in fermentation production.

In alcoholic beverage industries, ethanol generation is limited because some inhibitory compounds are generated during the fermentation process [14]. As a major inhibitory compound, acetic acid causes intracellular acidification and inhabits cell metabolism that acts to stress microbiota, ultimately leading to reduced product formation [15]. Previous studies have shown that non-*Saccharomyces* yeasts have a special capacity to deacidify products during fermentation process of alcoholic beverage industries [16,17]. For instance, non-*Saccharomyces* yeasts have the remarkable metabolic property to decrease concentration of acetic acid [18]. As a traditional spontaneous fermentation, there were *Saccharomyces* and multiple non-*Saccharomyces* yeasts to generate high quality and taste products in the Chinese *Maotai*-flavor *Baijiu* fermentation process [19]. The *Baijiu* production is often accompanied with a high concentration of acetic acid [9,20]. However, the tolerance mechanism of microbiota to acetic acid accumulation is not clear. Moreover, the pathway of acetic acid reduction involving key deacidifying non-*Saccharomyces* yeasts in microbiota is still unknown.

The aims of our study, to better understand the above problems, included (*i*) explore the influence of acetic acid accumulation to core functional yeasts involving *Saccharomyces* and multiple non-*Saccharomyces* yeasts in situ and in vitro; (ii) reveal the gene expressions related to acetic acid metabolism in *Saccharomyces* and non-*Saccharomyces* yeasts via metatranscriptomic sequencing; and (iii) illustrate the reduction pathway of acetic acid in non-*Saccharomyces* yeasts during Chinese *Maotai*-flavor *Baijiu* fermentation.

## 2. Materials and Methods 

### 2.1. Experimental Design and Sample Collection

The complete fermentation process was carried out in a natural environment with cereal materials (Figure S1). Sorghum was the main cereal material used in the *Baijiu* fermentation process. In addition, raw materials (almost 15 tons) were mixed with sorghum and wheat in a pit (3 m × 2.5 m × 4 m). The samples were collected in Zunyi City (27.42 N, 106.55 E), Guizhou Province, China. We selected a group of samples from a factory for Chinese *Maotai*-flavor *Baijiu* production. The fermentation process in pits usually lasted approximately 30 days, and three replicate samples were collected from the upper, middle, and bottom locations at five-day intervals (Figure S1). The samples from the different pit locations at day 5, 15, and 30 (three biological replicates, day 05-1, day 05-2, and day 05-3 for day 5; day 15-1, day 15-2, and day 15-3 for day 15; and day 30-1, day 30-2, and day 30-3 for day 30) were immediately pretreated to extract total RNA. Additional samples from the different pit locations were stored at −20 °C for metabolites analysis.

### 2.2. RNA Extraction, Metatranscriptomic Sequencing, and Analysis

The samples in situ for total RNA extraction were pretreated with sterile phosphate-buffered saline (PBS, 0.1 mol/L). Then, the samples were centrifuged at 70× *g* for 5 min to obtain the supernatant. After centrifugation, the supernatant was centrifuged at 12,000× *g* for 5 min to collect cells. The sediment was cooled and ground in liquid nitrogen and extracted with sodium laurate buffer (sodium laurate 10 g/L, Tris-HCl 0.1 mol/L, NaCl 0.1 mol/L, ethylenediaminetetraacetic acid (EDTA) 0.02 mol/L) with TRIzol (Sigma-Aldrich, St. Louis, MO, USA) to obtain total RNA. In addition, the metatranscriptomics libraries were constructed via the NEBNext^®^ Ultra™ RNA Library Prep Kit for Illumina (New England Biolabs, Ipswich, Ma). The ribosomal RNA was removed by Ribo-Zero™ rRNA Removal Kits (Bacteria) and Ribo-Zero™ Magnetic Gold Kits (Yeast) (Illumina, San Diego, CA, USA). Then, samples were quantified and sequenced on an Illumina Hiseq 2500 platform (Illumina, San Diego, CA, USA).

The raw reads from the metatranscriptomic gene sequencing were processed to obtain clean reads through the removal of ribosomal RNA sequences and low-quality reads (Q < 0.02) via Trimmomatic (version 0.36) [21]. The de novo transcriptomic assemblies were performed using the Trinity platform (version 2.40) [22]. We also input the results into MEGAN (version 5.11) [23] to obtain the species composition and relative abundance of information in the samples using DIAMOND (version 0.83) [24]. Then, we compared high-quality reads to a nonredundant protein database (Nr, NCBI) [25] and Kyoto Encyclopedia of Genes and Genomes (KEGG) [26] database to obtain functional annotation information using the basic local alignment search tool (BLAST, NCBI) software [27]. Details of the quality and assembly information about the metatranscriptomic data are shown in Tables S1 and S2. The metatranscriptomic data were submitted to NCBI SRA under accession number PRJNA377357.

### 2.3. Culture Fermentation 

The core yeast strains in *Baijiu* and culture fermentation were *Pichia kudriavzevii* C-16, *Saccharomyces cerevisiae* C-3, *Schizosaccharomyces pombe* C-11, and *Zygosaccharomyces bailii* C-7. In our study, four yeast species were isolated from *Baijiu* production and were identified via the forward primer ITS 1 (5′-TCCGTAGGTGAACCTGCGG-3′) and the reverse primer ITS 4 (5′-TCCTCCGCTTATTGATATGC-3′). Sorghum extract medium was used for mono- and co-culture fermentation [28]. We selected these yeast species (*P. kudriavzevii* C-16, *S. cerevisiae* C-3, *Schi. pombe* C-11, and *Z. bailii* C-7) to construct a synthetic consortium. They were pre-cultured in seed medium (sorghum extract medium) at 30 °C and 200 rpm for 48 h. Then, in mono-culture fermentation, 10% of the seed medium from one yeast strain was inoculated into the fermentation medium (sorghum extract medium) with an initial total strain density of about 2 × 10^6^ cells per milliliter. In co-culture fermentation, 10% of the seed medium from each yeast strains (totally 40% from four yeast species) was inoculated into the fermentation medium (sorghum extract medium) to form the synthetic consortium with an initial total strain density of about 8 × 10^6^ cells per milliliter. Mono- and co-culture fermentation were carried out with shake flasks. Uninoculated sorghum extract medium was also cultured as a negative control. The mono- and co-culture fermentations were all performed with 200 rpm at 30 °C for 72 h. All the experiments were performed in triplicate. According to the maximum concentration of acetic acid in the previous study [9], we created four sample groups in vitro with different conditions: in group +AA 10 g/L, co-culture fermentation (involved *P. kudriavzevii* C-16, *S. cerevisiae* C-3, *Schi. pombe* C-11, and *Z. bailii* C-7) or mono-culture fermentation (only *Schi. pombe* C-11) under initial concentration of 10 g/L acetic acid; in the none group, co-culture fermentation (involved *P. kudriavzevii* C-16, *S. cerevisiae* C-3, *Schi. pombe* C-11, and *Z. bailii* C-7) or mono-culture fermentation (only *Schi. pombe* C-11) without initial concentration of 10 g/L acetic acid.

### 2.4. Reverse-Transcription and Real-Time Quantitative PCR

Samples were collected from mono- and co-culture fermentations by centrifugation at 12,000× *g* at 4 °C for 5 min and the total RNA was extracted as follows: the samples were cooled and ground in liquid nitrogen and were transferred to a sterile Eppendorf tube, filled with 1.0 mL of precooled TRIzol reagent (Takara, Dalian, China). The ratios of A260/A280 and A260/A230 was calculated to assess RNA purity using a NanoDrop 8000 spectrophotometer (Thermo Scientific, Wilmington, DE, USA). The quality of the total RNA was analyzed by 1% nondenatured agarose gel electrophoresis. After that, the total RNA was immediately purified with 4 × gDNA wiper (Vazyme, Nanjing, China). Reverse transcription was conducted with 1 μg of total RNA sample in a 20 μL reaction mixture using the HiScript^®^ II QRT SuperMix for qPCR (+gDNA wiper) (Vazyme, Nanjing, China) to synthesize cDNA. All the operations followed the manufacturer’s instructions. Reactions without reverse transcriptase or a template were both used as negative controls. 

The microbial populations and gene transcription of different yeasts in mono- and co-culture fermentations were determined by real-time quantitative PCR. This quantitation was performed by a StepOnePlus real-time PCR system (Applied Biosystems, Foster City, CA, USA) with QuantiNova^®^ SYBR Green PCR Kit (Qiagen, Hilden, German). All the operations followed the manufacturer’s instructions. To quantify microbial populations in *P*. *kudriavzevii* C-16, *S*. *cerevisiae* C-3, *Schi. pombe* C-11, and *Z*. *bailii* C-7, we extracted total DNAs from samples in mono- and co-culture fermentations. All samples were pretreated with sterile phosphate-buffered saline (PBS, 0.1 mol/L). Then, the samples were centrifuged at 70× *g* for 5 min to obtain the supernatant. After centrifugation, the supernatant was centrifuged at 12,000× *g* for 5 min to collect microorganisms. After centrifugation, the sediment was cooled and ground in liquid nitrogen and extracted with sodium laurate buffer (sodium laurate 10 g/L, Tris-HCl 0.1 mol/L, NaCl 0.1 mol/L, ethylenediaminetetraacetic acid (EDTA) 0.02 mol/L) with phenol: chloroform: isoamyl alcohol (25:24:1) to obtain total DNA. High-quality total DNA was measured by 1% agarose gel electrophoresis and a NanoDrop 8000 Spectrophotometer (Thermo Scientific, Waltham, MA, USA) (260 nm/280 nm ratio). Genomic DNA samples were stored at −20 °C until used in additional procedures. All genomic DNAs and cDNAs were amplified in triplicate. The PCR mixtures comprised 10 μL 2 × QuantiNova SYBR Green RT-PCR Master Mix, 1 μL each of forward and reverse primer (1 μmol/L), and 1 μL of DNA template in a final volume of 20 μL. All qPCR runs were followed by preheating at 95 °C for 3 min, 40 cycles of 95 °C for 10 s, 60 °C for 30 s, and an increase of 0.5 °C every 5 s from 65 °C to 95 °C for melting curve analysis to confirm the specificity of the amplification. Negative controls were performed with no SYBR Premix Ex Taq, no template, or only double-distilled water. The primers used to amplify the microbial populations related to *P*. *kudriavzevii* C-16, *S*. *cerevisiae* C-3, *Schi. pombe* C-11, and *Z*. *bailii* C-7 are listed in Table 1. To quantify microbial populations in *P*. *kudriavzevii* C-16, *S*. *cerevisiae* C-3, *Schi. pombe* C-11, and *Z*. *bailii* C-7, ten-, and five-dilution series from four yeast genomic DNA were amplified by real-time quantitative PCR. To quantify expression genes in *P*. *kudriavzevii* C-16, *S*. *cerevisiae* C-3, *Schi. pombe* C-11, and *Z*. *bailii* C-7, the cycle threshold (CT) values of the replicates and reference genes (*UBC6*) were calculated. The genes acetyl-CoA hydrolase 1 (*ACH1*), acetyl-CoA C-acetyltransferase 1 (*ACCAT1*), acetyl-CoA synthetase 2 (*ACS2*), aldehyde dehydrogenase 5 (*ADH5*), hydroxymethylglutaryl-CoA synthase 1 (*HMGCS1*), hydroxymethylglutaryl-CoA reductase 1 (NADPH) (*HMGCR1*), and hexose transporter 1 (*HXT1*) were normalized to reference genes (*UBC6*). Finally, the relative expression of genes was quantified by the 2^−ΔΔ*C*T^ method [29]. The primers for above expression genes in *P*. *kudriavzevii* C-16, *S*. *cerevisiae* C-3, *Schi. pombe* C-11, and *Z*. *bailii* C-7 also were designed via the primer-BLAST tool [30] and are shown in Table 2.

### 2.5. Metabolites Analysis

The acetic acid of samples was identified with high-performance liquid chromatography (HPLC). All samples were pretreated with sterile PBS (0.1 mol/L). For the HPLC analysis, 4 g of material from each sample was taken and placed in 50 mL centrifuge tubes. Then, 30 mL of sterile, distilled water were added and mixed for 30 min in an ice-water bath. The HPLC analysis was performed with the same method as that in UPLC analysis. The HPLC system consisted of an Agilent 1200 HPLC (Agilent Technologies, Santa Clara, CA, USA) coupled to an Agilent 1200 Refractive Index Detector. The column was an Aminex 389 HPX-87H (300 mm × 7.8 mm, Bio-Rad, Hercules, CA, USA) column and the eluent was H_2_SO_4_. 

The ethyl acetate of samples was identified with headspace-solid phase microextraction-gas chromatography-mass spectrometry (HS-SPME-GC-MS). For metabolite analysis of ethyl acetate, 4 g of material from each sample was taken and placed in 50 mL centrifuge tubes. Then, 30 mL of sterile, distilled water and 0.3 g CaCl_2_ were added and mixed for 30 min in an ice-water bath. Finally, the supernatants were collected after centrifugation at 8000× *g* for 10 min. The automatic headspace sampling system (multipurpose sample MPS 2 with an adapter) for the solid-phase microextraction (SPME) (GERSTEL Inc., Baltimore, MD, USA) with 50/30 μm DVB/CAR/PDMS fiber (Supelco Inc., Bellefonte, PA, USA) were used for the SPME. The samples were preheated for 5 min and extracted for 45 min at 50 °C. An Agilent 6890N GC coupled with an Agilent 5975 mass selective detector (MSD) was used for gas chromatography-mass spectrometry (GC-MS). The GC-MS conditions were as follows: the starting temperature was 50 °C (held for 2 min), and it increased to 230 °C at a rate of 4 °C/min and was held at 230 °C for 15 min. 

The mevalonic acid of samples was also identified with GC-MS. During the fermentation process, mevalonic acid is easily to form mevalonolactone. A detailed description of mevalonic acid was recently described [32]. A 10 μL sample was injected using an Agilent GC 6890 N with an Agilent 5975 mass selective detector (MSD). The GC-MS conditions were as follows: The starting temperature was 90 °C (held for 1 min), and it increased to 250 °C at a rate of 30 °C/min and was held at 250 °C for 2 min. For absolute quantification, values of samples were fit to a generalized linear model generated from mevalonolactone standards.

### 2.6. Statistical Analysis

All statistical analyses and data plots were carried out with OriginPro (version 9.01), GraphPad Prism (version 8.02), Microsoft^®^ Excel, and Adobe Illustrator CS6. *P*-values were calculated with a nonparametric analysis in the Statistical Package for Social Science (SPSS, version 22.0) (Web Atlas, Paris, France).

## 3. Results

### 3.1. Potential Capacity of Non-Saccharomyces Yeast Schi. pombe in Response to Acetic Acid Stress

**Figure 1:**
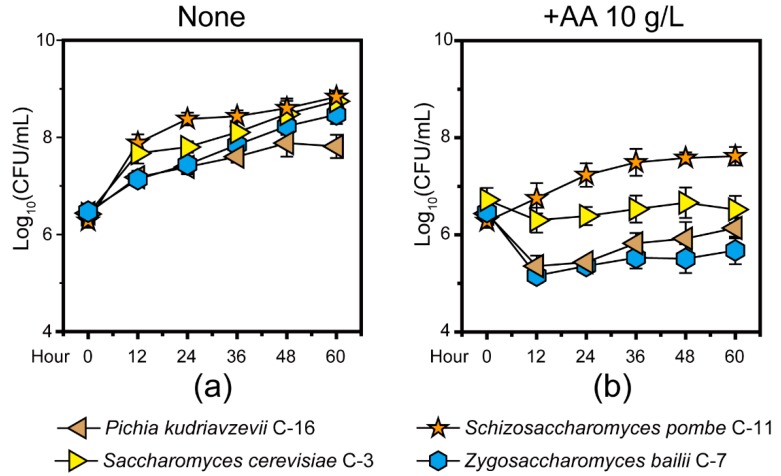
The microbial biomass of four core yeast strains (*Pichia kudriavzevii* C-16, *Saccharomyces cerevisiae* C-3, *Schizosaccharomyces pombe* C-11, and *Zygosaccharomyces bailii* C-7) isolated from fermentation microbiota in None (**a**) and +AA 10 g/L (**b**) group samples (see details in Section 2). Data are expressed as mean ± SD from biological triplicates. Error bars indicate standard deviation from three independent experiments.

**Figure 2:**
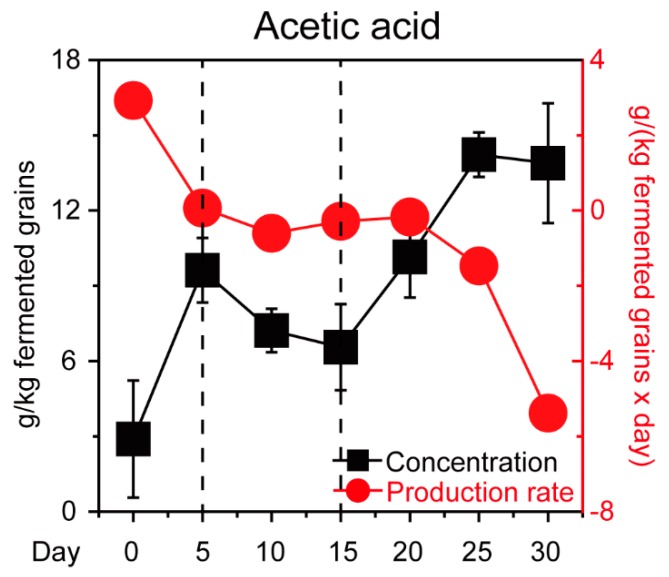
Dynamic concentration and production rate of acetic acid during *Baijiu* production. Data are expressed as mean ± SD from biological triplicates. Error bars indicate standard deviation from three independent sampling points.

In the previous study, we found *P. kudriavzevii*, *S. cerevisiae*, *Schi. Pombe,* and *Z. bailii* are the major functional contributors in Chinese *Maotai*-flavor *Baijiu* production [9]. Thus, we isolated and identified four core yeast strains that involved *S. cerevisiae* C-3 and three non-*Saccharomyces* yeast strains (*P. kudriavzevii* C-16, *Schi. pombe* C-11, and *Z. bailii* C-7) from fermentation microbiota. To illustrate how the four yeast strains tolerated acetic acid, we quantified the microbial populations in +AA 10 g/L and none group samples in vitro (Figure 1, see details in Materials and Methods). Compared with the none group samples, the population of four yeast strains showed a downward trend in the +AA 10 g/L group samples. With an initial concentration of about 10 g/L acetic acid, the population of *P. kudriavzevii* C-16 and *Z. bailii* C-7 showed a significant downward trend during the fermentation (Mann–Whitney *U* test, *p* < 0.001). Conversely, in the +AA 10 g/L group samples, the population of *Schi. pombe* C-11 showed an upward trend and reached a maximum of 3.82 × 10^7^ cells per milliliter at 48 h (Figure 1B). The growth curve analysis suggested that there was a significant apparent difference in growth of the four core yeasts between the none group and +AA 10 g/L group samples (Mann–Whitney *U* test, *p* < 0.001). However, the *Schi. pombe* C-11 samples exhibited clearly accelerated growth as compared to the other yeast strains in the samples from +AA 10 g/L group samples. Moreover, the acetic acid profiles in situ were qualified and quantified (Figure 2). The concentration of acetic acid decreased from the fifth day to day 15, with the average ranging from 9.62 g/kg fermented grains to 6.55 g/kg fermented grains. Moreover, the production rate of acetic acid decreased from day zero to 10, with the average ranging from 2.59 g/(kg fermented grains × day) to −0.36 g/(kg fermented grains × day). Although acetic acid accumulated during fermentation, the concentration of acetic acid decreased continuously, and the product rate remained unchanged substantially from the fifth day to day 15, during the fermentation process.

**Figure 3:**
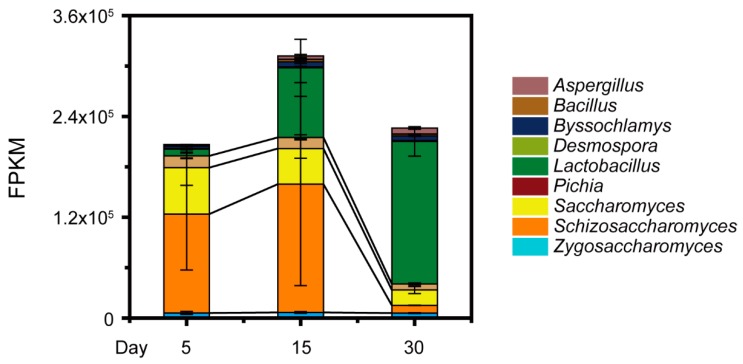
The gene expression of nine dominant fungal and bacterial genera (i.e., abundance >1%) by metatranscriptomic analysis on day 5, 15, and 30 during *Baijiu* production. The nine dominant genera are *Aspergillus*, *Bacillus*, *Byssochlamys*, *Desmospora*, *Lactobacillus*, *Pichia*, *Saccharomyces*, *Schizosaccharomyces,* and *Zygosaccharomyces*. Data are expressed as mean ± SD from biological triplicates. Error bars indicate standard deviation from three independent sampling points.

To further illustrate the potential mechanisms underlying acetic acid change in situ, a total of 59.12 G metatranscriptomic sequencing data were generated in the samples from pit A and pit B (4.93 ± 0.95 G per sample) (Tables S1 and S2). On the basis of the data accounting for 34.2% of the total metatranscriptomic (Fragments Per Kilobase per Million, FPKM), we observed nine core genera in the samples from the two pits (i.e., abundance >1%): *Aspergillus*, *Bacillus*, *Byssochlamys*, *Desmospora*, *Lactobacillus*, *Pichia* (major *P. kudriavzevii*), *Saccharomyces* (major *S. cerevisiae*), *Schizosaccharomyces* (major *Schi. pombe*), and *Zygosaccharomyces* (major *Z. bailii*) (Figure 3). The *Schizosaccharomyces* and *Lactobacillus* had high abundance of gene expression (748,663.11 FPKM and 781,4654.71 FPKM, respectively). Moreover, the abundance of gene expression related to core yeast *Schi. pombe* (368,058.68 FPKM) was much higher than that related to other core yeast species (187,219.88 FPKM) at day 15 during *Baijiu* production (Mann–Whitney U test, *p* < 0.001). These analyses demonstrated that non-*Saccharomyces* yeast *Schizosaccharomyces*, major *Schi. pombe*, could had direct correlation with the reduction of acetic acid during *Baijiu* production.

### 3.2. Schi. pombe Enhanced Conversion Efficiency of Acetic Acid to Generate Mevalonic Acid by Multiple Gene Upregulation

**Figure 4:**
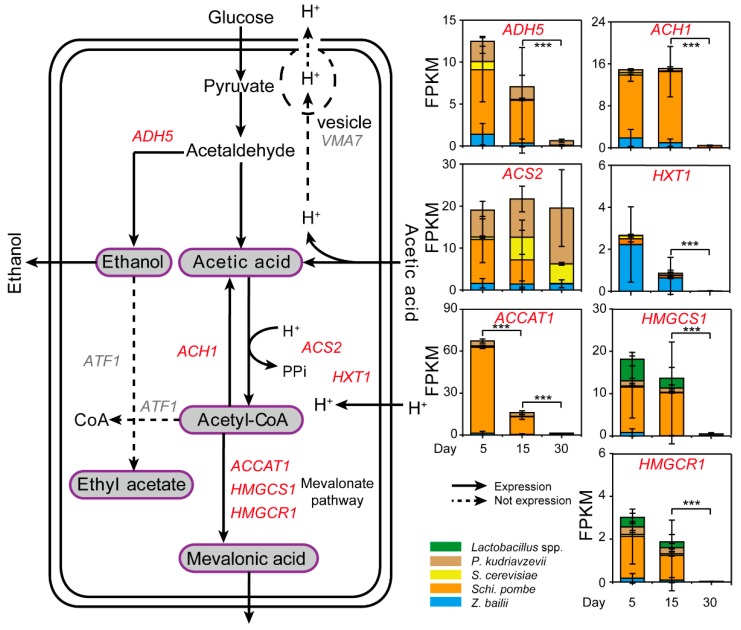
The differentially expressed genes of core yeast species (*P*. *kudriavzevii*, *S*. *cerevisiae*, *Schi*. *Pombe,* and *Z*. *bailii*) and *Lactobacillus* spp. related to acetic acid conversion on day 5, 15 and 30 during *Baijiu* production. According to the Kyoto Encyclopedia of Genes and Genomes (KEGG) database, these genes include alcohol dehydrogenase 5 (*ADH5*, K00121), acetyl-CoA hydrolase (*ACH1*, K01067), acetyl coenzyme A synthetase 2 (*ACS2*, K01895), hexose transporter (*HXT1*, K08139), V-type H+-transporting ATPase subunit F (*VMA7*, K02151), alcohol acetyltransferase 1 (*ATF1*), acetyl-CoA C-acetyltransferase 1 (*ACCAT1*, K00626), hydroxymethylglutaryl-CoA synthase 1 (*HMGCS1*, K01641), and hydroxymethylglutaryl-CoA reductase 1 (NADPH) (*HMGCR1*, K00021) in the core yeast species *P. kudriavzevii*, *S. cerevisiae*, *Schi. Pombe,* and *Z. bailii*. The genes related to different species are showed in different colors: *P. kudriavzevii* (brown), *S. cerevisiae* (yellow), *Schi. pombe* (orange) and *Z. bailli* (blue). Data are expressed as mean ± SD from biological triplicates. Error bars indicate standard deviation from three independent sampling points and asterisks represent significant differences between the two groups (*** *p* < 0.001, Mann–Whitney U test).

To explore the potential reduction mechanism of acetic acid, gene expressions relevant to the acetic acid generation and utilization were determined by metatranscriptomic analysis in situ (Figure 4 and Table S3). According to the Kyoto Encyclopedia of Genes and Genomes (KEGG) database, we found seven major expressed genes relevant to acetic acid reduction from the samples during *Baijiu* production. Among them, the gene *ACS2* (K01895) expressed at a high level during day five, 15, and 30 in situ (Figure 4). Expression of *ACS2* by *Schi. pombe* and *P. kudriavzevii* (average 10.44 and 6.40 FPKM, respectively) was observed in the samples, whereas the expression in *Z. bailii* and *S. cerevisiae* was 1.60 and 0.60 FPKM (on average) in the samples from day five. Conversely, expression of *HXT1* (K08139) by *Z. bailii* (average 2.23 FPKM) was observed in the samples from day five, whereas there was little expression in *S. cerevisiae* (average 0.27 FPKM) and *P. kudriavzevii* (average 0.01 FPKM). In addition, high expression of *ACCAT1* (K00626) was in *Schi. pombe* (average 61.58 FPKM), whereas there were few expressions in *Z. bailii* and *S. cerevisiae* (average 1.29 and 0.91 FPKM, respectively) in the samples from day five. Moreover, *Schi. pombe* had higher expression of *HMGCS1* (K01641, total 62.92 FPKM) and *HMGCR1* (K00021, total 9.37 FPKM) than other core yeast species and *Lactobacillus* spp. related to mevalonate pathway in the samples. These three genes also showed significant differences in expression between samples from day 15 and day 30 (Mann–Whitney U test, *p* < 0.001). However, there were no expression of *VMA7* (K02151) and *ATF1* (K00664). As shown in Figure S2A, the concentration of ethyl acetate decreased from the fifth day to day 15 with the average ranging from 0.78 g/kg fermented grains to 0.35 g/kg fermented grains. The concentration of ethyl acetate decreased with no expression of *ATF1* from day zero to 15 during fermentation. In addition, pH increased from day five to 15 with the average ranging from 3.65 to 3.67 (Figure S2B). Overall, the non-*Saccharomyces* yeast *Schi. pombe* reduced the concentration of acetic acid by enhancing the conversion efficiency from acetic acid to mevalonic acid during the fermentation process.

### 3.3. Response Mechanism of Schi. pombe to Acetic Acid Accumulation

**Figure 5:**
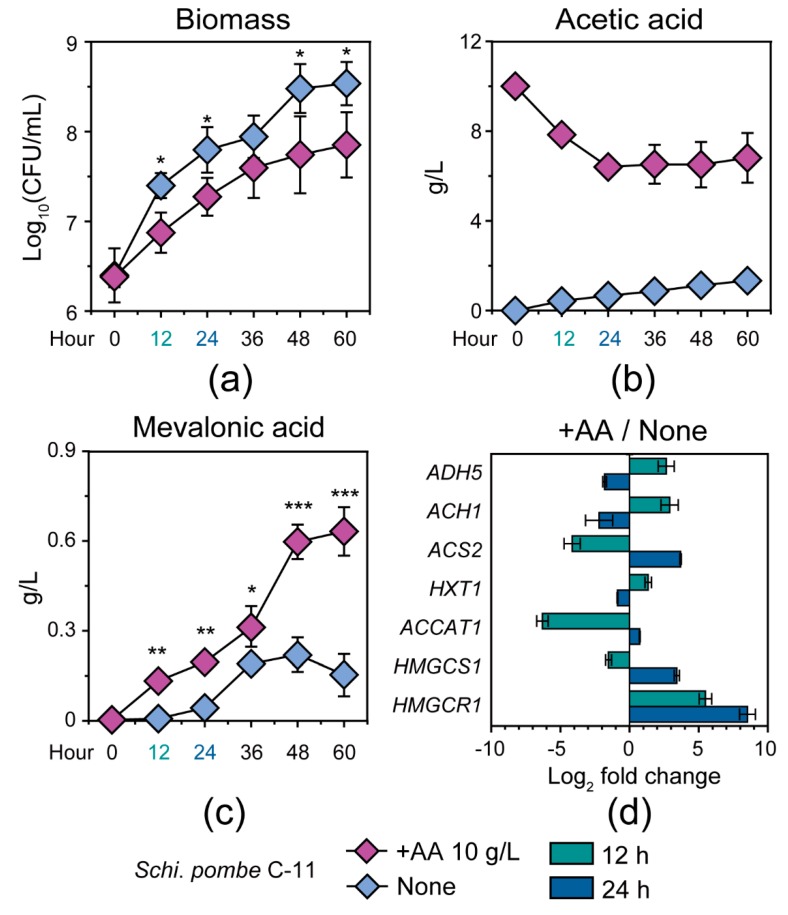
The microbial biomass, the metabolites concentration, and the gene expression of *Schizosaccharomyces pombe* C-11 in the +AA 10 g/L and none group samples in the mono-culture fermentation process. (**a**) The microbial biomass; (**b**,**c**) the concentration of acetic acid and mevalonic acid in the +AA 10 g/L and none group samples during the fermentation process; (**d**) the relative expression ratios of genes *ADH5*, *ACH1*, *ACS2*, *HXT1*, *ACCAT1*, *HMGCS1,* and *HMGCR1* in +AA 10 g/L group as compared to the none group at 12 h and 24 h. In the +AA 10 g/L group samples, the mono-culture process using *Schi. pombe* C-11 under initial concentration of 10 g/L acetic acid; and in the none group samples, the mono-culture process using *Schi. pombe* C-11 without initial concentration of 10 g/L acetic acid. The length in each square frame represents relative gene expression ratio based on real-time quantitative PCR at 12 h and 24 h, respectively. Data are expressed as mean ± SD from biological triplicates. Error bars indicate standard deviation from three independent experiments and asterisks represent significant differences between the two groups (* *p* < 0.05, ** *p* < 0.01, *** *p* < 0.001, Mann–Whitney U test).

To test and verify the conversion ability of *Schi. pombe* from acetic acid to mevalonic acid in *Baijiu* production, we identified and quantified the populations of *Schi. pombe* C-11 in the +AA 10 g/L and none group samples in mono-culture fermentation (see details in Section 2) (Figure 5). As shown in Figure 5A, the population of *Schi. pombe* C-11 showed a strong bias between the samples from the +AA 10g/L and the none groups (Figure 5A). *Schi. pombe* C-11 reached a maximum population of 1.15 × 10^9^ cells per milliliter at 60 h in the + AA 10 g/L group samples, whereas *Schi. pombe* C-11 reached a maximum population of 4.05 × 10^9^ cells per milliliter at 60 h in the none group samples (Figure 5A). In addition, the concentration of acetic acid showed a downward trend and reached a minimum of 6.40 ± 0.18 g/L at 24 h from +AA 10 g/L samples with the initial concentration of acetic acid about 10 g/L in mono-culture fermentation (Figure 5B). Interestingly, the concentration of mevalonic acid had a significant change from the +AA 10 g/L group samples. In comparison to the samples of the none group, mevalonic acid showed a distinct upward trend from 36 h to 60 h (Figure 5C, Mann–Whitney *U* test, *p* < 0.001). Moreover, we also explored the transcription levels of related genes (*ADH5*, *ACH1*, *ACS2*, *HXT1*, *ACCAT1*, *HMGCS1,* and *HMGCR1*) at 12 h and 24 h in the samples from the +AA 10 g/L and none groups (Figure 5D). As compared with culture fermentation of no added acetic acid, the transcription of *ADH5* (average from 2.65 to −1.80, log_2_ fold change), *ACH1* (average from 2.90 to −2.20, log_2_ fold change) and *HXT1* (average from 1.35 to −0.87, log_2_ fold change) was decreased from 12 h to 24 h in the +AA 10 g/L group. Moreover, the transcription levels of genes related to acetic acid utilization were upregulated, including *ACS2* (average from −4.15 to 3.69, log_2_ fold change), *ACCAT1* (average from −6.30 to 0.73, log_2_ fold change), *HMGCS1* (average from −1.52 to 3.42, log_2_ fold change), and *HMGCR1* (average from 5.48 to 8.53, log_2_ fold change) in *Schi. pombe* C-11 from 12 h to 24 h in the +AA 10 g/L group samples.

## 4. Discussion

Chinese *Maotai*-flavor *Baijiu* production is a traditional spontaneous fermentation process with various non-*Saccharomyces* yeasts and multiple flavor-related products [6]. It provides a good opportunity to explore the functions of non-*Saccharomyces* yeasts in microbiota [5,33]. We have previously reported that non-*Saccharomyces* yeasts have important effects on the concentration of acetic acid [9]. Moreover, previous reports have also demonstrated that non-*Saccharomyces* yeasts could moderate acetic acid with final concentrations below 0.3 g/L [18,34]. Thus, discovering the characteristics of non-*Saccharomyces* yeasts could help to illustrate concentration changes of acetic acid during *Baijiu* production.

In our work, a high concentration of acetic acid inhibited the growth of core functional yeasts that involved one *Saccharomyces* and three non-*Saccharomyces* yeast strains (Figure 1). In addition, one unanticipated finding was that the concentration of acetic acid decreased in the samples from the fifth day to day 15 and non-*Saccharomyces* yeast *Schi. pombe* had a direct correlation with the reduction in situ (Figure 2 and Figure 3). Although multiple *Lactobacillus* species have great acetic acid productivity, they have no ability to degrade the concentration of acetic acid in *Baijiu* production [9,14]. Conversely, *Schi. pombe* has great acetic acid productivity and could produce low levels of acetic acid with other non-*Saccharomyces* yeasts [18]. As compared with previous reports, our findings suggested that *Schi. pombe* could convert acetic acid to other metabolites although the microbial biomass is limited by the stress of acetic acid. The high-level expressions of gene *ACCAT1*, *HMGCS1,* and *HMGCR1* explained that microbiota reduces the concentration of acetic acid in the cell through the mevalonate pathway in *Schi. pombe* instead of ethyl acetate synthesis in *S. cerevisiae*, thereby improving microbiota to tolerate the stress of acetic acid in situ (Figure 4). It was reported that the undissociated form of acetic acid can enter the yeast cells by simple diffusion (pKa > pH_ext_) or through the *S. cerevisiae* Fps1p [35]. To maintain neutral pH in the cytoplasm, the hydrogen ion (H^+^) dissociated from acetic acid can be excluded via vacuolar efflux from intracellular to extracellular, such as *VMA7* [36]. Intracellular acetate ion (CH_3_COO−) dissociated from acetic acid can also be metabolized through the conversion to acetyl-CoA [37,38]. Then, acetyl-CoA in the cell could product ethyl acetate by alcohol acetyltransferases (e.g., ATF1) with ethanol to tolerate acetic acid stress in *S. cerevisiae* [39]. Thus, the formation of ethyl acetate is an effective way to reduce acetic acid [40]. However, our findings assumed that the *Schi. pombe* uses a new way to convert acetic acid and improves the reduction capacity under acid stress.

To test and verify the abovementioned hypothesis, we cultivated the strain *Schi*. *pombe* C-11 under different acid conditions in vitro. The results showed that acetic acid inhibited the growth of *Schi*. *pombe* C-11 in the + AA 10 g/L group samples as compared with the none group (Figure 5, see details in Materials and Methods). Moreover, *Schi. pombe* C-11 improved the conversion efficiency of acetic acid to generate mevalonic acid in the + AA 10 g/L group samples, which could reduce the concentration of acetic acid and relieve its inhibition in fermentation microbiota (Figure 5). There are several reasons why the microbiota chose the mevalonate pathway to reduce acetic acid by *Schi. pombe* C-11 in Chinese *Maotai*-flavor *Baijiu* production. These reasons include: (*i*) The first possible explanation is that efflux H^+^ from the cell cannot solve the problem of intracellular low pH. The extracellular H^+^ would pass through the membrane again into the cells, thereby energy stored in the cells is continuously consumed. With the fermentation process, yeast cells cannot maintain their normal pH range, eventually causing yeast to lack their functions [41]. (*ii*) In the case of low pH in cell, *ATF* gene (optimum pH ≈ 7) was inhibited and its function was lacking in *S*. *cerevisiae* [42]. Thus, *S*. *cerevisiae* cannot reduce acetic acid by forming ethyl acetate [42]. (*iii*) As a non-*Saccharomyces* yeast, *Schi*. *pombe* has no metabolic pathway related to *ATF* gene. However, *Schi*. *pombe* has mevalonate pathway to generate terpenes from acetic acid, thereby reducing the stress of acetic acid ultimately in microbiota [43,44]. Therefore, studies need to explore further information about the microbial interactions between *S*. *cerevisiae* and non-*Saccharomyces* yeasts at the molecular level.

In conclusion, we revealed the function of non-*Saccharomyces* yeast *Schi*. *pombe* in fermentation microbiota based on culture-independent and -dependent methods. By converting acetic acid to form mevalonic acid, *Schi. pombe* reduced the concentration of acetic acid to improve acid resistance in fermentation microbiota. In addition, *Schi. pombe* also generated the precursor of terpenes synchronously to enhance the quality and taste of Chinese *Maotai*-flavor *Baijiu*. These findings enriched our knowledge about a new metabolic mechanism of non-*Saccharomyces* yeasts in acetic acid utilization. Our study also provided reference approaches to decode the functions of non-*Saccharomyces* yeasts in various alcoholic beverage industries.

## Figures and Tables

**Table 1:** Primers used for microbial population analysis in four yeast strains.

Primer Name	Sequence (5′-3′)	Primer Size (bp)	Reference
PkF	GTTTGAGCGTCGTTTCCATC	20	[31]
PkR	AGCTCCGACGCTCTTTACAC	20	
ScF	GTGCGCGGTCTTGCTAGGCT	20	this work
ScR	TACCTCTGGGCCCCGATTGC	20	
SpF	AGTGAAGCGGGAAAAGCTCA	20	this work
SpR	ATCGACCAAAGACGGGGTTC	20	
ZbF	CATGGTGTTTTGCGCC	16	[8]
ZbR	CGTCCGCCACGAAGTGGTAGA	21	

**Table 2:** Primers used for gene expression analysis in *Schi. pombe*.

Primer Name	Sequence (5′-3′)	Primer Size (bp)	Reference
ACH1SpF	TGCATACACCATCGGTTCGT	20	this work
ACH1SpR	GAGCACGTTCGGTAGGAGAC	20	
ACCAT1SpF	GCTTCTCTTCCTGCCACCAA	20	this work
ACCAT1SpR	TGGCCAAGATTGGCTGAGAC	20	
ACS2SpF	AGAGTCTGTTGCAGACCGTG	20	this work
ACS2SpR	CATACTGGCGGTAGGCTCAG	20	
ADH5SpF	AGTTGGATCCATGGGTGCTT	20	this work
ADH5SpR	TTCCGGTTTCGCTTCAGCAT	20	
HMGCS1SpF	GTGGCGTGAACGCTCTTTTT	20	this work
HMGCS1SpR	GGGGCATTAGGACCAACCAA	20	
HMGCR1SpF	AGAGGTCGGCAATTGGACTG	20	this work
HMGCR1SpR	AGTGCGAGCGATCAACTGAA	20	
HXT1SpF	GCCGGTACTGTGAAAAGGGA	20	this work
HXT1SpR	TCAGTTTGGATTGATGCGCTG	21	
UBC6SpF	TTGGCTGTTGCCATCCTTTG	20	this work
UBC6SpR	GGAAACGTCCGCTTGGAGTA	20	

## Supplementary Materials

The following are available online at https://www.mdpi.com/2076-2607/7/12/606/s1, Figure S1: Fermentation process, Figure S2: Dynamic concentration of ethyl acetate and pH, Table S1: Metatranscriptomic sequencing quality information, Table S2: Metatranscriptomic sequencing assembly information, Table S3: KEGG expression genes.
