# Supplementary material for: Schizosaccharomyces pombe Can Reduce Acetic Acid Produced by Baijiu Spontaneous Fermentation Microbiota
Source: Microorganisms. 2019 Nov 22;7(12):606. doi: 10.3390/microorganisms7120606 (PMC6956116; doi:10.3390/microorganisms7120606)
Supplement: Supplementary file 1 [file microorganisms-07-00606-s001.zip › Supplemental Material.docx]

# Supplemental Data


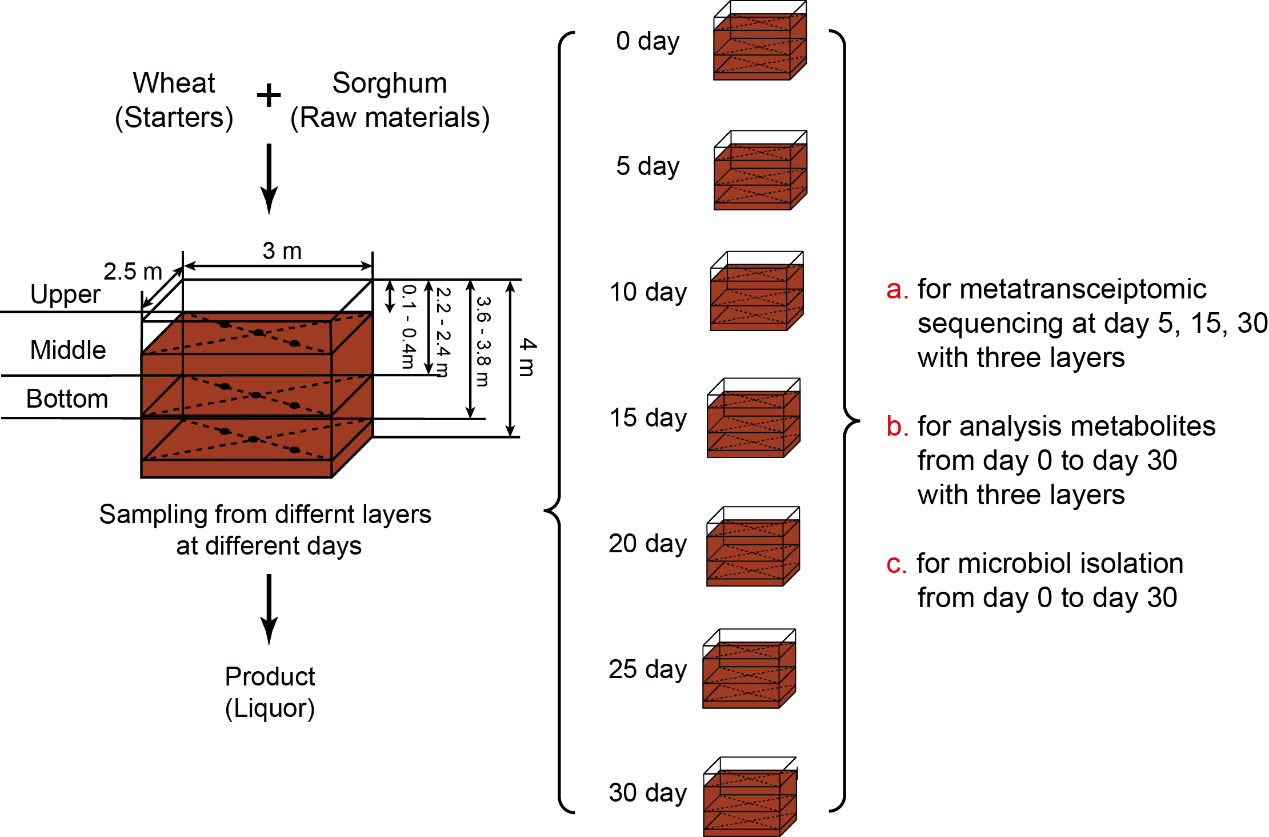
**Figure S1.** Chinese *Maotai*-flavor *Baijiu* production. The samples were collected from the upper, middle and bottom layer in pit fermentation respectively. In addition, sample in the same layer was made by mixing samples from three points for reducing the volatility of samples.


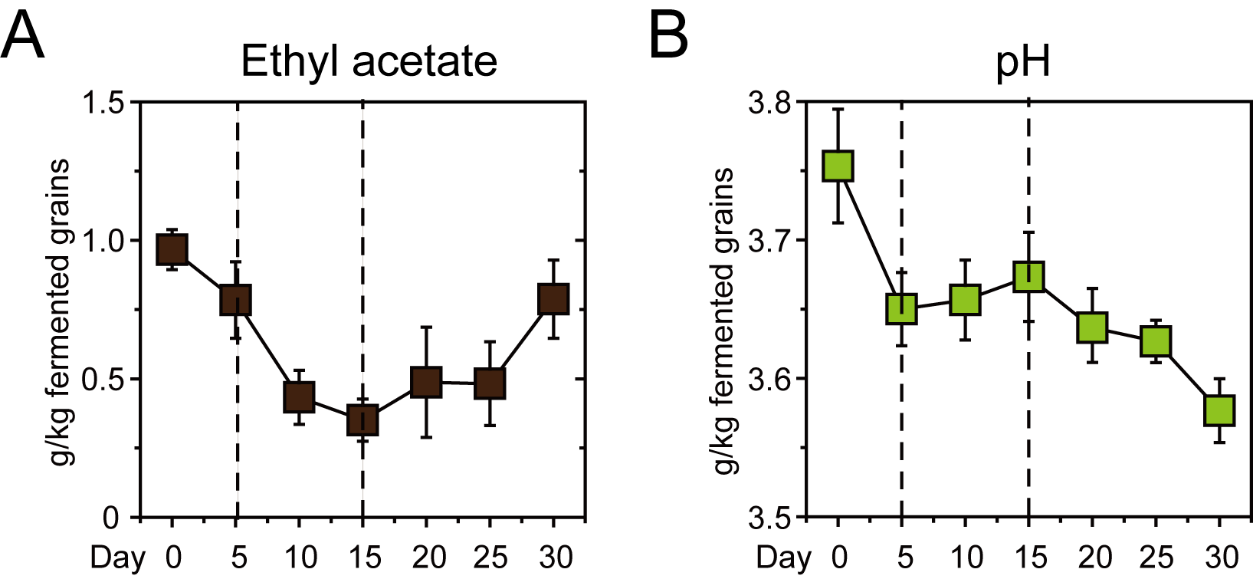
**Figure S2.** Dynamic concentration of ethyl acetate and pH during *Baijiu* production. Data are expressed as mean ± SD from biological triplicates.

**Table S1.** Summary of the metatranscriptomic sequencing quality information across samples.

| **Samples**  **(n = 9)** | **Base Sum** | **Clean Reads** | **Q20 (%)** | **Q30 (%)** | **GC (%)** |
| --- | --- | --- | --- | --- | --- |
| Day 05-1 | 4,404,399,779 | 17,484,963 | 95.86 | 89.87 | 48.74 |
| Day 05-2 | 3,623,578,464 | 14,390,297 | 96.64 | 90.40 | 48.52 |
| Day 05-3 | 3,808,745,532 | 15,127,713 | 96.65 | 90.51 | 90.51 |
| Day 15-1 | 4,034,867,046 | 16,020,821 | 98.01 | 93.15 | 44.47 |
| Day 15-2 | 3,306,219,618 | 13,397,321 | 98.24 | 96.42 | 47.82 |
| Day 15-3 | 746,911,934 | 3,016,156 | 97.67 | 95.29 | 52.20 |
| Day 30-1 | 5,129,371,014 | 20,369,457 | 98.42 | 93.79 | 43.60 |
| Day 30-2 | 5,264,823,386 | 21,067,685 | 98.91 | 97.40 | 43.13 |
| Day 30-3 | 4,683,423,338 | 18,890,622 | 96.40 | 90.96 | 52.81 |

**Table S2**. Summary of the metatranscriptomic sequencing assembly information across samples.

| **Samples**  **(n = 9)** | **rRNA**  **Reads** | **rRNA**  **percent (%)** | **Contig**  **number** | **Total**  **length (bp)** | **Largest**  **length (bp)** | **N50**  **(bp)** |
| --- | --- | --- | --- | --- | --- | --- |
| Day 05-1 | 120,767 | 0.69 | 76,850 | 85,968,562 | 16,195 | 1,264 |
| Day 05-2 | 176,198 | 1.22 | 23,806 | 22,020,239 | 10,388 | 953 |
| Day 05-3 | 182,107 | 1.20 | 27,240 | 23,942,843 | 10,902 | 884 |
| Day 15-1 | 265,993 | 1.66 | 37,310 | 54,651,466 | 18,338 | 1,910 |
| Day 15-2 | 1,023,654 | 3.82 | 8,844 | 9,498,462 | 16,134 | 1,784 |
| Day 15-3 | 184,813 | 3.06 | 5,140 | 4,661,114 | 7,748 | 921 |
| Day 30-1 | 480,982 | 2.36 | 2,806 | 4,235,311 | 20,664 | 2,170 |
| Day 30-2 | 420,176 | 1.00 | 3,557 | 5,103,099 | 18,661 | 1,888 |
| Day 30-3 | 800,540 | 2.12 | 2,907 | 4,539,766 | 13,621 | 2,205 |
